# Feasibility and Acceptability of Oura Ring Sleep Monitoring in Older Adults Admitted From the Emergency Department: Prospective Cohort Study

**DOI:** 10.2196/92359

**Published:** 2026-09-30

**Authors:** Adrian D Haimovich, Annie Lacourciere, Kerry Palihnich, Sydney Mulqueen, Natalie Jansen, Suzanne M Bertisch, Mara A Schonberg, Aanand D Naik, Sarah D Berry, Edward R Marcantonio, Nathan I Shapiro, Janet M Mullington

**Affiliations:** 1 Department of Emergency Medicine, Beth Israel Deaconess Medical Center, 330 Brookline Avenue, Boston, MA, 02215, United States, 1 617-667-7001; 2 Department of Medicine, Beth Israel Deaconess Medical Center, Boston, MA, United States; 3 Department of Medicine, Brigham and Women’s Hospital, Boston, MA, United States; 4 Department of Management, Policy and Community Health, School of Public Health, and Division of Geriatrics and Palliative Medicine, McGovern Medical School, University of Texas Health Science Center at Houston (UTHealth Houston), Houston, TX, United States; 5 Department of Neurology, Beth Israel Deaconess Medical CenterBoston, MA, United States

**Keywords:** emergency department, sleep hygiene, wearables, delirium, older adults

## Abstract

**Background:**

Emergency department (ED) boarding exposes admitted older adults to a high-disruption environment. Sleep loss during acute hospitalization is associated with adverse outcomes and may contribute to delirium, yet objective sleep measurement in this setting is difficult. Consumer multisensor wearables may permit low-burden, longitudinal measurement, but the feasibility and acceptability of ring-based monitoring among acutely ill older adults is unknown.

**Objective:**

This study aimed to estimate the feasibility and acceptability of Oura Ring Gen 3 sleep monitoring in older adults admitted to the hospital from the ED, and to describe exploratory device-measured and patient-reported sleep outcomes.

**Methods:**

We conducted a single-site prospective cohort study from February through March 2025. Patients aged 65 years or older with an inpatient medical bed request were recruited in the ED and asked to wear the ring continuously for up to 4 days or until discharge; monitoring continued after transfer to an inpatient unit from the ED. Participants underwent daily 3-Minute Diagnostic Interview for Confusion Assessment Method-Defined Delirium (3D-CAM) assessments, cognitive testing when not delirious, daily sleep surveys, and an exit survey. Feasibility outcomes were the approached-to-enrolled proportion and the proportion of study completers with wear for more than 75% of the analyzed period. Acceptability was the proportion of exit respondents who disagreed or strongly disagreed that the ring bothered them. Device-estimated sleep duration was normalized to hours per 24 hours, and its association with mean patient-reported sleep quality was assessed using Kendall τ.

**Results:**

Of 29 patients approached for consent, 15 enrolled (51.7%, 95% CI 32.5%‐70.6%); 1 withdrew immediately, leaving 14 who completed study procedures. Of these 14 patients, 13 wore the ring for ≥75% of their enrollment period (92.9%, 95% CI 66.1%‐99.8%). Eleven participants completed the exit survey, and 9 reported that the ring was not bothersome (81.8%, 95% CI 48.2%‐97.7%). Among the 13 participants who wore the ring for ≥75% of the analyzed period, median device-estimated sleep was 4.3 (IQR 2.6-7.0) hours per 24 hours. Participants reported good or very good sleep on 9 of 19 surveyed nights (47.4%). Mean sleep quality and device-estimated sleep duration were positively associated among 10 participants with complete paired data (Kendall τ=0.54; *P*=.04).

**Conclusions:**

In this pilot study, we found that sleep monitoring with the Oura Ring Gen 3 was feasible and acceptable to older adults admitted from the ED. These findings support larger studies validating the Oura Ring against polysomnography in hospitalized older adults.

## Introduction

Sleep-wake disturbances are associated with adverse cardiometabolic derangements and poor health outcomes during acute inpatient admissions, including delirium [1-4]. Hospital admissions lead to frequent sleep interruptions due to environmental factors (eg, noise and light), patient conditions (eg, pain), and clinical care routines (eg, phlebotomy and vital-sign checks) [3,5-8]. In a recent study, only around 5% of admitted older adults had an uninterrupted sleep window of at least 7 hours [5].

Inpatient boarding, the practice of holding admitted patients in the ED while they wait for an inpatient bed, is increasingly common, with more than 30% of admitted older adults waiting longer than 3 hours [9]. An overnight stay in the ED has been associated with higher inpatient mortality among older adults, particularly those who need support with activities of daily living [10]. Contemporary ED delirium management emphasizes reducing avoidable disruptions and promoting sleep when possible [11]. Compared with older adults already on inpatient units, those remaining in the ED experience more sleep interruptions and shorter sleep windows [5]. These observations have largely relied on patient reports or care-event timing rather than continuous objective sleep measurement.

Polysomnography, an overnight sleep study recording multiple physiological variables, is widely considered the criterion standard for sleep assessment [12]. Wearable devices, including wrist actigraphs, are lower-burden alternatives that have been used in the inpatient setting [13-15]. More recently, the Oura Ring, a multisensor ring device, has demonstrated agreement with polysomnography for sleep detection and staging in ambulatory studies [16,17]. It has also been evaluated alongside other wearables in free-living neurologic research [18]. The Oura Ring uses accelerometry, temperature, photoplethysmography (measurement of blood volume changes in microvascular tissue beds), and circadian features in proprietary sleep algorithms [19]. Recent independent work suggests that older adults with mild cognitive impairment or dementia can participate in longitudinal wearable sleep research outside the hospital [20]. However, feasibility and acceptability remain under-studied among acutely hospitalized older adults, where cognitive impairment, delirium, transfers between units, clinical procedures, and device removal may impede data collection.

To address this gap, the primary aim of this prospective cohort study was to estimate recruitment feasibility, device-wear feasibility, and acceptability of Oura Ring Gen 3 sleep monitoring among older adults admitted to the hospital from the ED. Secondary aims were to describe device-estimated sleep duration, patient-reported sleep quality and interruptions, and the association between device-estimated sleep duration and patient-reported sleep quality.

## Methods

### Study Design and Setting

This was a single-site prospective cohort study conducted at the Beth Israel Deaconess Medical Center (BIDMC) ED in Boston, Massachusetts. Study recruitment occurred from February through March 2025.

### Participants and Recruitment

Participants aged 65 years or older with an ED disposition to an inpatient medical service were eligible. Inclusion criteria were (1) age 65 years or older, (2) an inpatient bed request placed between 1 PM and 10 PM, and (3) admission to a medical rather than surgical service. Exclusion criteria were (1) critical illness or admission to an intensive care unit; (2) inability to read or communicate verbally in English, including hearing or visual impairment that precluded study procedures; (3) an active major psychiatric condition, such as psychosis or bipolar disorder; and (4) current heavy alcohol use.

Recruitment was limited to weekdays from 1 PM to 10 PM, when research staff were available. During each staffed period, a study team member screened the electronic health record (EHR) for patients with an inpatient medical bed request, reviewed available records against the eligibility criteria, and approached eligible patients in the ED when clinical care permitted. Patients were followed for up to 4 hospitalization days or until discharge, whichever occurred first; the ED encounter date was considered study day 1.

A study team member explained the study purpose and procedures and assessed decisional capacity before obtaining written informed consent. Patients without decisional capacity could enroll through a legally authorized representative (LAR). Monitoring began after enrollment in the ED and continued through transfer to an inpatient medical unit.

### Oura Ring Measures

Participants were provided an Oura Ring Gen 3 (Oura Health Ltd), a consumer multisensor wearable that uses accelerometry, photoplethysmography, and temperature sensors to estimate sleep and physiologic measures [19] (Figure 1). The ring was intended to be worn continuously during the study period and was returned to the study team at the conclusion of study procedures.

**Figure 1.**
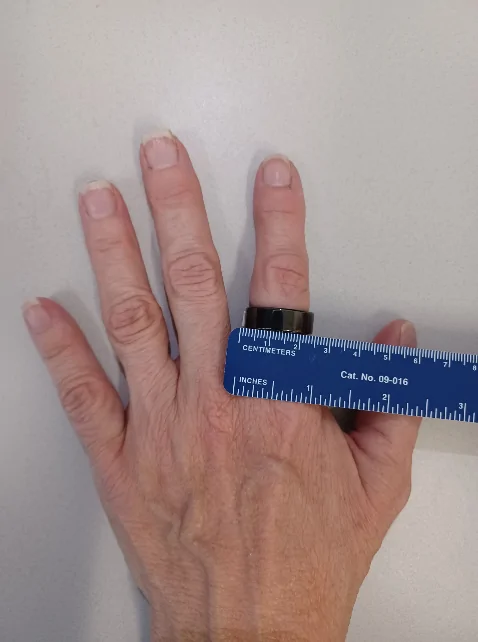
Oura Ring Gen 3 used in the study. Photo of research team member included with consent.

Each ring was synced directly to an Apple iPhone SE provided by the study team. No protected health information was included during ring registration. Data were exported from the Oura Teams platform as comma-separated value files. One-minute metabolic equivalent of task (MET) data were used to estimate wear time; the use of Oura MET data for energy-expenditure assessment has been evaluated previously [21]. Sleep outcomes were obtained from the exported Oura sleep records.

### Study Procedures

After consent, participants underwent the 3-Minute Diagnostic Interview for Confusion Assessment Method-Defined Delirium (3D-CAM) and completed a demographic survey that included a medication review for sleep-related medications, such as trazodone [22]. The 3D-CAM was subsequently administered daily. Participants with delirium either at enrollment or during the subsequent evaluations remained eligible for device monitoring but were not asked to complete daily surveys while delirious.

After the first overnight period, participants without delirium completed the Montreal Cognitive Assessment (MoCA) [23]. Each day, participants rated the prior night’s sleep on a 5-point Likert scale from very poor to very good and completed the PROMIS (Patient-Reported Outcomes Measurement Information System) Sleep Disturbance short form [24].

Participants also identified any causes of sleep interruption, selecting all applicable options from noise, pain, light, tests such as x-ray, blood work, medication administration, room changes, nurses or technicians obtaining vital signs, other causes, or no interruption.

On study days 2 and 3, research staff asked the clinical team whether discharge was expected that day. If discharge was expected, or if the participant had reached study day 4, a research assistant administered the exit survey. Acceptability was assessed with the statement, “This ring bothered me while participating in this study,” rated on a 5-point Likert scale from strongly disagree to strongly agree. Participants with delirium at the time of planned exit survey administration did not complete the survey.

Research assistants reviewed the EHR for documented dementia, cognitive impairment, insomnia, and sleep apnea. Demographic, chart-review, and survey data were stored in a BIDMC REDCap database [25].

### Outcome Measures

The primary recruitment feasibility outcome was the proportion of patients approached who consented to participate. The primary device-wear feasibility outcome was the proportion of participants completing study procedures whose device wear percentage exceeded 75% of the analyzed period. The primary acceptability outcome was the proportion of exit survey respondents who disagreed or strongly disagreed with the statement that the ring bothered them. Secondary outcomes were device-estimated hours of sleep per 24 hours, daily self-reported sleep quality, sleep disturbance questionnaire responses, and patient-reported causes of sleep interruption.

### Data Analysis

Continuous variables were summarized with medians and IQRs, and categorical variables were summarized with counts and percentages. Recruitment feasibility, device-wear feasibility, and acceptability proportions were reported with 2-sided 95% Clopper-Pearson exact binomial CIs.

Wear-time analysis was anchored at 9 PM on the calendar day of the first available MET record. A minute was classified as worn when the MET value was 0.9 or greater. Device cessation was defined as the first minute after which all remaining MET values were less than 0.9. Wear percentage was calculated within each complete 24-hour interval from 9 PM to 9 PM and averaged across available intervals for each participant. A participant met the device-wear feasibility criterion when this percentage exceeded 75%. ED and inpatient data were analyzed together, as the exact moment of patient movement between settings was not available.

For participants who met the device-wear criterion, the analyzable sleep window began at 9 PM on the calendar day of the first available MET record and ended at estimated device cessation. For sleep records overlapping a boundary, total sleep duration was attributed in proportion to the fraction of the sleep-record interval within the analyzable window. Total attributed sleep was divided by elapsed analyzable time and multiplied by 24 to obtain hours slept per 24 hours. For the association analysis, daily sleep-quality ratings were averaged within each participant and compared with participant-level device-estimated sleep duration using the Kendall τ. All tests were 2-sided, with *P*<.05 considered statistically significant, and no adjustment was made for multiple comparisons because the secondary analyses were exploratory.

No formal sample size calculation was performed because this pilot study was intended to estimate feasibility and acceptability during the planned recruitment period. Accordingly, emphasis was placed on CIs and descriptive estimates rather than hypothesis testing. Oura Ring and survey data were analyzed using Python (version 3.11) with *pandas*, *NumPy*, and *SciPy*.

### Ethical Considerations

The BIDMC institutional review board approved the study (2024P000977).

### Trial Registration

This prospective observational cohort did not assign an intervention and was not registered as a clinical trial.

## Results

### Participant Flow and Characteristics

During the recruitment period, 29 patients were approached for consent. Fifteen enrolled, including 2 through LAR consent. Among the 14 who did not enroll, 9 declined, 2 lacked capacity and their LARs declined, and 3 lacked capacity without an available LAR. One participant withdrew immediately after enrollment, resulting in 14 participants who completed study procedures and were included in the feasibility analysis (Figure 2).

**Figure 2.**
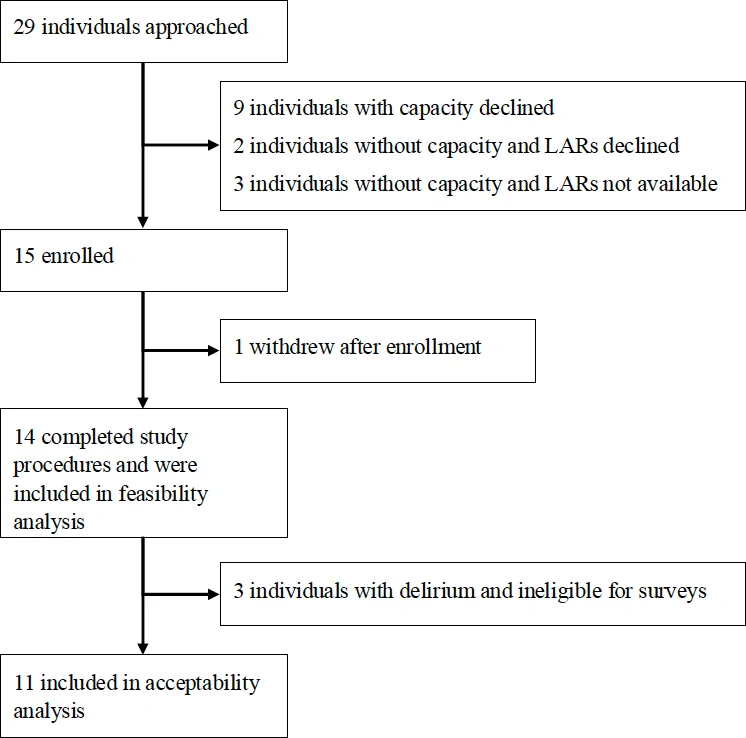
Participant flow diagram. LAR: legally authorized representative.

Participant characteristics are shown in Table 1. The median age was 78 (IQR 75‐82; range 65‐92) years, and 7 participants were female. One participant identified as Black and non-Hispanic and 13 participants identified as White and non-Hispanic. Three participants, all with delirium, did not complete the MoCA; among the remaining 11 participants, the median score was 20 (IQR 18‐22). Three participants had documented dementia or cognitive impairment, 4 had obstructive sleep apnea, none had documented insomnia, and 4 reported using a sleep medication at home. Two participants had delirium at enrollment, and 2 additional participants developed delirium during the study. The median number of monitored nights per participant was 2 (IQR 1‐3).

**Table 1.** Participant characteristics among participants included in the feasibility analysis (n=14).

Characteristics	Value
Age (years), median (IQR; range)	78.0 (75.3‐81.8; 65‐92)
Female sex, n (%)	7 (50.0)
MoCA^a^ score (n=11), median (IQR)	20 (18‐22)
Married, n (%)	7 (50.0)
Current smoker, n (%)	2 (14.3)
Prior diagnosis of sleep or cognitive disorder, n (%)	
Cognitive impairment or dementia	3 (21.4)
Obstructive sleep apnea	4 (28.6)
Insomnia	0 (0.0)
Home sleep medication use, n (%)	4 (28.6)
Delirium at enrollment, n (%)	2 (14.3)
Incident delirium, n (%)	2 (14.3)
Study nights, median (IQR)	2 (1-3)

a MoCA: Montreal Cognitive Assessment.

### Feasibility and Acceptability

The recruitment feasibility estimate was 15 of 29 approached patients (51.7%, 95% CI 32.5%‐70.6%). Of the 14 participants who completed study procedures, 13 had wear data for more than 75% of the analyzed period (92.9%, 95% CI 66.1%‐99.8%). The median participant-level wear percentage was 93.4% (IQR 91.0%‐96.2%). The 1 participant who did not meet the wear criterion had delirium at enrollment and wore the ring for approximately 8% of the analyzed period.

Eleven participants completed the exit survey; 3 did not complete it because of delirium at the planned assessment. One of these participants was also transferred to the intensive care unit, and the ring was removed. Of the 11 exit survey respondents, 9 strongly disagreed or disagreed that the ring bothered them, for an acceptability estimate of 81.8% (95% CI 48.2%‐97.7%). Five strongly disagreed, 4 disagreed, 1 was neutral, and 1 strongly agreed.

### Sleep Outcomes

Across 19 completed daily surveys, participants rated their sleep as good or very good on 9 nights (47.4%). Only 3 nights (15.8%) were reported as having no interruption. The most frequently reported interruptions were vital-sign collection on 13 nights (68.4%), blood work on 12 nights (63.2%), noise on 8 nights (42.1%), and medication administration on 6 nights (31.6%; Table 2).

**Table 2.** Patient-reported causes of sleep interruption across 19 surveyed nights. Participants could select more than one interruption.

Sleep interruption	Nights, n (%)
Noise	8 (42.1)
Pain	1 (5.3)
Light	1 (5.3)
Going to tests (eg, x-ray)	2 (10.5)
Blood work	12 (63.2)
Medication administration	6 (31.6)
Changing rooms	2 (10.5)
Nurses or technicians obtaining vital signs	13 (68.4)
Other	3 (15.8)
Nothing interrupted sleep	3 (15.8)

Among the 13 participants who met the device-wear criterion, median device-estimated sleep was 4.3 (IQR 2.6-7.0) hours per 24 hours. Among 10 participants with complete paired device and survey data, mean patient-reported sleep quality had a moderate positive association with device-estimated sleep duration (Kendall τ=0.54; *P*=.04).

## Discussion

In this single-site pilot study, 13 of 14 participants who completed study procedures met the prespecified device-wear criterion, and 9 of 11 exit survey respondents reported that the ring was not bothersome. The participant who did not meet the wear criterion had delirium at enrollment. All 3 participants without exit survey data had delirium at the planned assessment and were therefore excluded from the acceptability denominator; 1 of these patients was also transferred to the intensive care unit. Taken together, these findings suggest that ring-based monitoring is feasible and acceptable but that delirium may complicate device and study retention.

These findings extend prior work on lower-burden sleep monitoring in hospitalized patients [13,14]. Recent research has also shown that older adults with mild cognitive impairment or dementia can complete longitudinal wearable sleep monitoring outside the hospital [20]. Our results suggest that a ring device can be retained during acute hospitalization by most participants who complete study procedures, despite advanced age, cognitive impairment, and movement between the ED and inpatient units.

Both the Oura data and patient reports indicated substantial barriers to hospital sleep, in alignment with inpatient studies identifying care routines, noise, and light as common disruptions [3,5-7]. The median 4.3 hours of sleep per 24 hours was lower than the pooled estimate of approximately 5.8 hours reported for hospitalized older adults in a prior meta-analysis [6]. Possible explanations include differences in measurement method, the inclusion of time spent in the ED, the advanced age and cognitive vulnerability of the cohort, and the high frequency of clinical interruptions. Moreover, we observed a moderate association between patient-reported sleep quality and device-estimated duration, providing preliminary evidence of concordance between the measures.

This study has several limitations. First, the sample was small, producing imprecise feasibility and acceptability estimates. Second, recruitment occurred at a single academic medical center during staffed weekday afternoon and evening periods, and the English-language and communication requirements may limit generalizability. Third, 3 participants with delirium did not complete the exit survey, creating potential selection bias in the acceptability estimate. Fourth, sleep estimates were not compared concurrently with polysomnography or research-grade actigraphy, and the proprietary algorithm was not developed specifically for acutely hospitalized older adults. Fifth, the wear-time method relied on a MET threshold and 9 PM–anchored complete intervals, and sleep duration was normalized to analyzable participation time; these analytic choices may differ from other definitions of wear and sleep opportunity. Sixth, ED and inpatient-unit time were pooled because unit-location timestamps were unavailable, so the study cannot isolate sleep during ED boarding.

Among older adults admitted from the ED who completed study procedures, Oura Ring Gen 3 monitoring was feasible for most participants and was not bothersome to most exit survey respondents. Larger multisite studies should use location-aware monitoring, include concurrent polysomnography or research-grade actigraphy in a validation subset, and develop retention strategies for patients with delirium or cognitive impairment. This work could establish whether low-burden multisensor wearables can support pragmatic measurement of hospital sleep and the evaluation of interventions intended to reduce sleep disruption.

## Abbreviations

3D-CAM: 3-Minute Diagnostic Interview for Confusion Assessment Method-Defined Delirium
BIDMC: Beth Israel Deaconess Medical Center
ED: emergency department
EHR: electronic health record
LAR: legally authorized representative
MET: metabolic equivalent of task
MoCA: Montreal Cognitive Assessment
PROMIS: Patient-Reported Outcomes Measurement Information System
